# Microtubule and Actin Differentially Regulate Synaptic Vesicle Cycling to Maintain High-Frequency Neurotransmission

**DOI:** 10.1523/JNEUROSCI.1571-19.2019

**Published:** 2020-01-02

**Authors:** Lashmi Piriya Ananda Babu, Han-Ying Wang, Kohgaku Eguchi, Laurent Guillaud, Tomoyuki Takahashi

**Affiliations:** ^1^Cellular and Molecular Synaptic Function Unit, Okinawa Institute of Science and Technology (OIST) Graduate University, Okinawa 904–0495, Japan, and; ^2^Molecular Neuroscience group, Institute of Science and Technology Austria (IST Austria), Am Campus 1, A-3400 Klosterneuburg, Austria

**Keywords:** F-actin, microtubules, neurotransmission, short-term depression, synaptic vesicles

## Abstract

Cytoskeletal filaments such as microtubules (MTs) and filamentous actin (F-actin) dynamically support cell structure and functions. In central presynaptic terminals, F-actin is expressed along the release edge and reportedly plays diverse functional roles, but whether axonal MTs extend deep into terminals and play any physiological role remains controversial. At the calyx of Held in rats of either sex, confocal and high-resolution microscopy revealed that MTs enter deep into presynaptic terminal swellings and partially colocalize with a subset of synaptic vesicles (SVs). Electrophysiological analysis demonstrated that depolymerization of MTs specifically prolonged the slow-recovery time component of EPSCs from short-term depression induced by a train of high-frequency stimulation, whereas depolymerization of F-actin specifically prolonged the fast-recovery component. In simultaneous presynaptic and postsynaptic action potential recordings, depolymerization of MTs or F-actin significantly impaired the fidelity of high-frequency neurotransmission. We conclude that MTs and F-actin differentially contribute to slow and fast SV replenishment, thereby maintaining high-frequency neurotransmission.

**SIGNIFICANCE STATEMENT** The presence and functional role of MTs in the presynaptic terminal are controversial. Here, we demonstrate that MTs are present near SVs in calyceal presynaptic terminals and that MT depolymerization specifically prolongs the slow-recovery component of EPSCs from short-term depression. In contrast, F-actin depolymerization specifically prolongs fast-recovery component. Depolymerization of MT or F-actin has no direct effect on SV exocytosis/endocytosis or basal transmission, but significantly impairs the fidelity of high-frequency transmission, suggesting that presynaptic cytoskeletal filaments play essential roles in SV replenishment for the maintenance of high-frequency neurotransmission.

## Abstract (precis)

Cytoskeletal filaments such as microtubules (MTs) and filamentous actin (F-actin) dynamically support cell structure and functions. In central presynaptic terminals, F-actin is expressed along the release edge and reportedly plays diverse functional roles, but whether axonal MTs extend deep into terminals and play any physiological role remains controversial.

## Introduction

The cytoskeleton, comprising filamentous actin (F-actin), microtubules (MTs), and intermediate filaments, supports the cell architecture and functions. During neuronal development, MTs and F-actin play crucial roles in cell motility, axonal growth, and organelle/protein transport to growth cones (Arnette et al., 2016). However, their functional roles in developed synapses remain unestablished. Among cytoskeletal elements, F-actin is localized at the distal end of presynaptic axons (Hirokawa et al., 1989; Saitoh et al., 2001). Functionally, it is thought to regulate neurotransmitter release (Morales et al., 2000), mediate endocytosis of synaptic vesicles (SVs; Watanabe et al., 2013; Delvendahl et al., 2016; Wu et al., 2016), and promote the recovery of synaptic responses from activity-dependent short-term depression (STD; Cole et al., 2000; Sakaba and Neher, 2003) via fast SV replenishment (Lipstein et al., 2013) and clearance of used SVs from release sites (Hosoi et al., 2009; Lee et al., 2012).

Compared with F-actin, much less is known about the presynaptic functional roles of MTs, except that they are involved in the transport of mitochondria and presynaptic elements such as those of active zones (AZs) or SVs (Hirokawa et al., 2010; Melkov and Abdu, 2018). Previous electron microscopy (EM) studies at the frog neuromuscular junction (NMJ) reported that MTs anchoring SVs are directing toward AZs (Gray 1978, 1983; Hirokawa et al., 1989). Likewise, at the *Drosophila* NMJ, the MT-associated protein Futsch (Hummel et al., 2000) links MT to AZs, thereby supporting neurotransmitter release (Lepicard et al., 2014). However, at lamprey or chick embryonic synapses, MTs do not colocalize with SVs (Smith et al., 1970; Bird, 1989). At the calyx of Held in adult cats, MTs are observed in presynaptic terminal swellings, but not in the SV pool (Perkins et al., 2010). In a recent imaging study in cultured calyceal presynaptic terminals, MTs are shown to be present within terminal swellings and depolymerization of MTs by nocodazole treatment impaired long-distance SV movements between swellings, whereas depolymerization of F-actin had no effect (Guillaud et al., 2017). However, in cultured hippocampal synapses, interbouton SV trafficking is reportedly blocked by an F-actin depolymerizing drug (Darcy et al., 2006).

To address whether MTs play any functional role in presynaptic terminals, we first examined their localization in the calyx of Held of rodent brainstem using confocal and stimulated emission depletion (STED) microscopy. We then depolymerized MTs in slices, using vinblastine, and examined whether it affects synaptic functions. For comparisons, we also depolymerized F-actin using latrunculin A, and re-examined its effects on synaptic functional properties (Sakaba and Neher, 2003), newly at physiological temperature (PT; 37°C) and at calyces of Held in posthearing rats. Our results revealed differential contributions of F-actin and MTs, specifically to fast and slow recovery of EPSCs from STD without cross talk, suggesting that these cytoskeletal elements independently contribute to recycling of SVs to maintain high-frequency neurotransmission at this fast synapse.

## Materials and Methods

All experiments were performed in accordance with guidelines of the Physiological Society of Japan and animal experiment regulations at Okinawa Institute of Science and Technology Graduate University.

### 

#### 

##### Slice preparation and solutions for electrophysiological recordings.

Brainstems were isolated from Wistar rats of either sex, at the age of postnatal day 13–16, after decapitation under isoflurane anesthesia. Transverse brainstem slices (175–250 μm in thickness), containing the medial nucleus of the trapezoid body (MNTB), were cut using a vibroslicer (VT1200S, Leica) in ice-cold solution containing the following (mm): 200 sucrose, 2.5 KCl, 26 NaHCO_3_, 1.25 NaH_2_PO_4_, 6 MgCl_2_, 10 glucose, 3 *myo*-inositol, 2 sodium pyruvate, and 0.5 sodium ascorbate, at pH 7.4, when bubbled with 95% O_2_ and 5% CO_2_, and 310–320 mOsm. Before recordings, slices were incubated for 1 h at 37°C in artificial CSF (aCSF) containing the following (mm): 125 NaCl, 2.5 KCl, 1 MgCl_2_, 2 CaCl_2_, 10 glucose, 3 *myo*-inositol, 2 sodium pyruvate, 0.5 sodium ascorbate, 1.25 NaH_2_PO_4_, and 26 NaHCO_3_, at pH 7.4, when bubbled with 95% O_2_ and 5% CO_2_, and 310–315 mOsm. For recording EPSCs, the aCSF contained 10 μm bicuculline methiodide (Sigma-Aldrich) and 0.5 μm strychnine hydrochloride (TCI) to block GABA_A_ and glycine receptors, respectively. The pipette solution for recording postsynaptic currents contained the following (mm): 110 CsF, 30 CsCl, 10 HEPES, 5 EGTA, 1 MgCl_2_, and 5 QX314-Cl, at pH 7.3–7.4, adjusted with CsOH, and 300–320 mOsm, unless otherwise noted. For presynaptic membrane capacitance measurements, the aCSF contained 10 mm tetraethylammonium chloride (TCI), 0.5 mm 4-aminopyridine (Nacalai Tesque), 1 μm tetrodotoxin (Nacalai Tesque), 10 μm bicuculline methiodide, and 0.5 μm strychnine hydrochloride. The pipette solution for presynaptic membrane capacitance (*C*_m_) measurements contained the following (mm): 105 Cs gluconate, 30 CsCl, 10 HEPES, 0.5 EGTA, 12 disodium phosphocreatinine, 3 Mg-ATP, 0.3 Na_2_-GTP, and 1 MgCl_2_, at pH 7.3–7.4, adjusted with CsOH, and 315–320 mOsm. For simultaneous presynaptic and postsynaptic action potential (AP) recording, the presynaptic pipette solution contained the following (mm): 110 potassium gluconate, 30 KCl, 5 EGTA, 12 disodium phosphocreatine, 10 l-glutamate, 1 MgCl_2_, 3 Mg-ATP, and 0.3 Na_2_-GTP, at pH 7.3–7.4, adjusted with KOH, and 315–320 mOsm. The postsynaptic pipette solution contained the following (mm): 120 potassium gluconate, 30 KCl, 5 EGTA, 12 disodium phosphocreatine, 1 MgCl_2_, 3 Mg-ATP, 0.3 Na_2_-GTP, and 1 l-arginine, at pH 7.3–7.4 adjusted with KOH, 315–320 mOsm). To depolymerize MTs, slices were incubated for 20–60 min at PT (35–37°C) with vinblastine sulfate (50 μm; Wako) dissolved in aCSF. To depolymerize F-actin, slices were incubated with latrunculin A (20 μm; Wako) at PT for 60 min.

##### Fluorescence imaging.

For fixed tissue imaging, the following primary antibodies were used: Anti-VGluT1 guinea pig antiserum (1:2000; AB5905, Millipore; RRID:AB_2301751); anti-synaptophysin rabbit polyclonal antiserum (1:250; catalog #101002, Synaptic Systems; RRID:AB_887905); anti-α-tubulin mouse monoclonal clone DM1A (1:250; catalog #T9026, Sigma-Aldrich; RRID:AB_477593); and anti-β3-tubulin rabbit antiserum (1:1000; product #2200, Sigma-Aldrich; RRID:AB_262133). Secondary antibodies were goat IgG conjugated with Invitrogen Alexa Fluor 488, 568, or 647 (Thermo Fisher Scientific). For fixed tissue imaging, acute brainstem slices (250 μm) were cut (see above) and fixed with 4% paraformaldehyde in PBS for 30 min at 37°C and overnight at 4°C. On the following day, slices were rinsed three times in PBS, permeabilized in PBS containing 0.5% Triton X-100 (Tx-100; Nacalai Tesque) for 30 min and blocked in PBS containing 3% bovine serum albumin (BSA; Sigma-Aldrich) and 0.05% Tx-100 for 45 min. Slices were incubated overnight at 4°C with primary antibody diluted in PBS 0.05% Tx-100, 0.3% BSA. On the next day, slices were rinsed three times with PBS containing 0.05% Tx100 for 10 min and incubated with corresponding secondary antibody diluted in PBS 0.05% Tx-100, 0.3% BSA for 1 h at room temperature (RT). Slices were further rinsed three times in PBS 0.05% Tx-100 for 10 min and finally were washed in PBS for another 10 min. Before mounting, the nucleus was stained with Life Technologies NucBlue (Thermo Fisher Scientific) in PBS for 20 min according to manufacturer instruction. For confocal imaging, slices were then directly mounted on glass slides (Matsunami) using a mounting medium (Ibidi) and sealed using nail polish. For STED microscopy, slices were sequentially incubated in PBS containing 10%, 20%, and 50% of 2,2′-thiodiethanol (TDE) for 1 h each, followed by washing three times in 97% TDE solution for 10 min each, and mounted on glass slides using TDE mounting reagent (Abberior).

For live imaging of silicon-rhodamine (SiR)-tubulin-stained slices, acute brainstem slices (250 μm) were incubated with SiR-tubulin (1 μm; Cytoskeleton) at 37°C according to manufacturer instructions. The slices were then mounted onto a 35 mm Ibidi dish and immobilized using a platinum grid holder, then incubated in aCSF containing 50 μm vinblastine at 37°C. HeLa cells, cultured in DMEM 10% FBS and grown in a 35 mm Ibidi dish for 3 d, were incubated with 1 μm SiR-tubulin or SiR-actin at 37°C and 5% CO_2_. Culture medium was replaced with Tyrode's solution before vinblastine or latrunculin A treatment and observation.

Confocal images were acquired on a laser scanning microscope (LSM 780, Carl Zeiss) equipped with a Plan-apochromat 63×, oil-immersion objective with a numerical aperture (NA) of 1.4 and excitation laser lines (wavelengths: 405, 488, 561, and 633 nm). For quantifying fluorescence intensity levels, the region of interest was delimited to calyceal terminals, and background fluorescence was subtracted using ImageJ software. Super-resolution imaging was performed on STED 3× TCS SP8 microscope (Leica) equipped with an HC PL APO CS2 100×, 1.4 NA oil-immersion objective (Leica) and tunable white laser excitation line, and with depletion laser lines (wavelengths: 592, 660, and 775 nm). STED images were deconvoluted using Huygens software (Leica). Three-dimensional reconstruction of STED 3× confocal stacks was performed in Imaris 9.2 with filament tracer and measurement pro plugins (Bitplane/Oxford Instruments) to estimate the distance between identified vesicles and microtubules.

##### Electrophysiological recordings and data analysis.

The calyx of Held presynaptic terminals and postsynaptic MNTB principal cells were visually identified with a 40× water-immersion objective attached to an upright microscope (BX51WI, Olympus). All experiments were performed at PT. EPSCs were evoked in MNTB principle neurons by afferent fiber stimulation using a bipolar tungsten electrode, positioned on the axon bundles halfway between the midline and MNTB region. For whole-cell recording of EPSCs, MNTB principal neurons were voltage clamped at a holding potential of −70 mV. Postsynaptic pipettes were pulled for a resistance of 2–3 MΩ and had a series resistance of 4–10 MΩ, which was compensated by 40–70% for a final value of 3 MΩ. STD of EPSCs was induced by a train of 30 stimuli at 100 Hz, and recovery from STD was monitored from EPSCs evoked by stimulations at different intervals (0.02–30 s). The recovery time course was fit by a double exponential function, from which fast time constants (τ_fast_) and slow time constants (τ_slow_) were measured.

Presynaptic membrane capacitance measurements at calyces of Held were made as previously described (Sun et al., 2002; Yamashita et al., 2005) except that recordings were made at PT in the present study instead of RT. Calyceal terminals were voltage clamped at a holding potential of −80 mV, and single-pulse step to +10 mV (20 ms in duration) was applied for inducing presynaptic Ca^2+^ currents. The rate of endocytosis was evaluated from the 50% decay time of membrane capacitance change.

##### Data analysis.

Electrophysiological data were acquired at a sampling rate of 50 kHz using an EPC-10 patch-clamp amplifier controlled by PatchMaster software (HEKA), after on-line filtering at 5 kHz and subsequently analyzed off-line using IGOR Pro 6.22 (WaveMetrics), Excel 2010 (Microsoft), Origin Pro 8.6 (Origins Laboratory), SPSS (IBM), and Prism 6 (GraphPad Software). Data fitting was performed using the least-squares method (single or double exponential). Imaging data were analyzed using Las AF Lite (Leica), ZEN (Zeiss), and ImageJ. All values are given as the mean ± SEM, and 95% confidence intervals on the difference of the means were considered statistically significant by two-tailed unpaired *t* test or one-way ANOVA with a *post hoc* Scheffé's test (*p* < 0.05).

## Results

### Colocalization of microtubules with SVs at the calyx of Held presynaptic terminals

**Figure 1.**
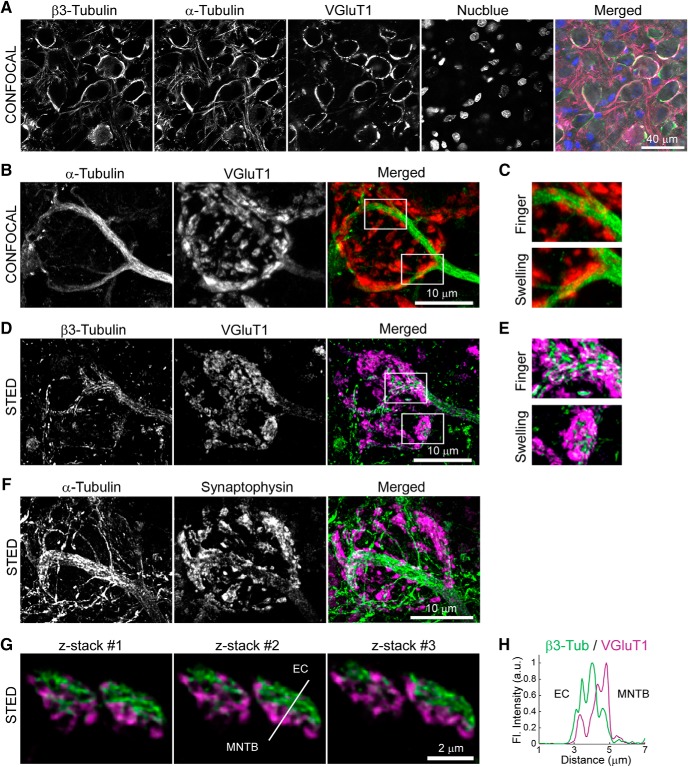
Localization of microtubules in the calyx of Held presynaptic terminals. ***A***, Confocal images of β3-tubulin, α-tubulin, VGluT1, and Nucblue in the MNTB region of postnatal day 15 brainstem slice. ***B***, Confocal images of α-tubulin and VGluT1 immunofluorescence, and their merged image in a calyx of Held presynaptic terminal. ***C***, Zoom-in of the area delineated in ***B*** (white boxes) showing MTs (green) and SVs (red) in presynaptic fingers and swellings. ***D***, High-resolution STED images of β3-tubulin and VGluT1 immunofluorescence and their merged image in a calyx of Held presynaptic terminal. ***E***, Zoom-in of the area delineated in ***D*** (white boxes) showing colocalization of MTs (green) and SVs (magenta) in presynaptic fingers and swellings. ***F***, High-resolution STED image of α-tubulin and synaptophysin in the calyx of Held presynaptic terminal. ***G***, High-resolution STED *z*-stack (*z* = 300 nm) images of presynaptic swellings immunolabeled with β3-tubulin (green) and VGluT1 (magenta). ***H***, Line-scan profile of the fluorescence (Fl.) signal intensity in arbitrary unit (a.u.) along the line on ***G*** from the extracellular (EC) side to the postsynaptic (MNTB neuron) side, showing partial overlap of MTs immunolabeled with β3-tubulin (β3-Tub, green) and SVs immunolabeled with VGluT1 (magenta).

**Figure 2.**
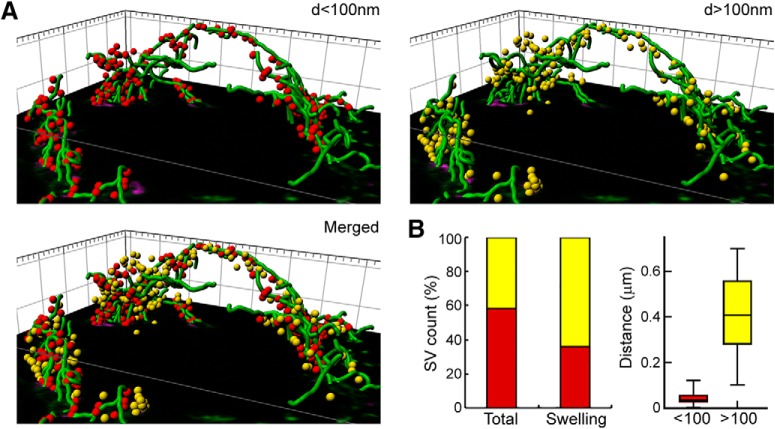
3D reconstruction of SVs and MTs in a presynaptic terminal. ***A***, 3D reconstruction of STED confocal stacks from Figure 1, *D* and *G*, showing MTs (green) and SVs (red, distance (d) < 100 nm, left; yellow, *d* > 100 nm, right; merged, bottom left). ***B***, Bar graphs for the percentage of SVs localized at <100 nm (red) or >100 nm (yellow) from MTs, in the whole terminal (left) and in four swellings in the terminal (right). Box chart of the average distance estimated between SVs and MTs in presynaptic swelling for the two groups of SVs (red, <100 nm; yellow, >100 nm; *n* = 106 SVs for each group). Error bars indicate ± SEM.

Before addressing the functional roles of MTs in presynaptic terminals, we examined the localization of MTs in the calyx of Held presynaptic terminals in the brainstem of juvenile rats (postnatal day 13–16) using confocal and STED microscopy. After tissue fixation and permeabilization to washout-free tubulin, immunofluorescence staining of calyceal terminals, using specific antibodies against α- or β3-tubulin, revealed tubulin-polymer bundles, running along axons and extending into calyceal terminals surrounding postsynaptic MNTB neurons (Fig. 1*A–C*). Using STED, tubulin-polymer bundles were clearly observed in proximity with SVs labeled with vesicular glutamate transporter 1 (VGluT1, Fig. 1*D*,*E*) or synaptophysin (Fig. 1*F*). At high magnifications, immunofluorescent tubulin signals were seen to partially overlap with those of SVs labeled with VGluT1 (Fig. 1*G*,*H*). Three-dimensional reconstruction of STED confocal stacks and analyses of SV distance from MTs indicated that 59% of SVs are localized on or alongside the MT lattice within 100 nm in the whole terminal (Fig. 2*A*,*B*). In presynaptic swellings, 36% of SVs were localized within 100 nm of MTs with an average ± SEM distance of 44 ± 2.5 nm (*n* = 106 SVs), whereas 64% of SVs were distributed >100 nm away from MTs with an average distance of 408 ± 16 nm (*n* = 106; Fig. 2*B*). These results indicate that MTs are extended and spread into calyceal swellings and suggest that MTs are partially colocalized with a subset of glutamatergic SVs in the nerve terminal.

### Monitoring depolymerization of microtubules in presynaptic terminals in slices

**Figure 3.**
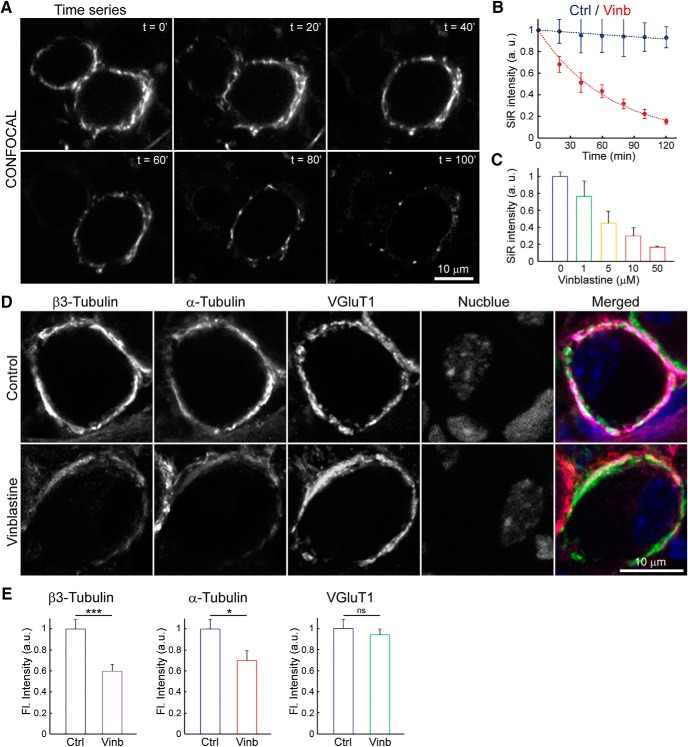
Depolymerization of microtubules in calyx of Held presynaptic terminals. ***A***, Real-time imaging of MTs labeled with SiR-tubulin in live slices, indicating progressive depolymerization before (*t* = 0) and during (*t* = 20–100 min) vinblastine treatment (50 μm) at PT. ***B***, Time plots showing normalized SiR-tubulin fluorescence intensity with (red) or without (blue) vinblastine treatment (50 μm) from three independent experiments (*n* = 9 cells from 3 slices of 3 animals). ***C***, Bar graphs showing the dose-dependent effect of vinblastine on SiR-tubulin fluorescence intensity after 2 h of treatment at PT (*n* = 3 cells). ***D***, Confocal images of calyces of Held terminals in fixed slices immunolabeled with β3-tubulin, α-tubulin, and VGluT1, without (top panels) or after (bottom panels) vinblastine (50 μm) treatment for 60 min at PT. ***E***, Quantification of fluorescence intensity of β3-tubulin [control (Ctrl), *n* = 20 from 3 slices of 3 animals; vinblastine (Vinb), *n* = 23; *p* = 0.0008], α-tubulin (Ctrl, *n* = 20; Vinb, *n* = 23; *p* = 0.023), and VGluT1 (Ctrl, *n* = 31; Vinb, *n* = 28; *p* = 0.589), without treatment (Ctrl) or after vinblastine treatment (Vinb). Unpaired Student's *t* test: *p < 0.05; ***p < 0.001. ns, Not significant; Ctrl, control; Vinb, vinblastine. Error bars indicate ± SEM.

To address the functional roles of MTs in presynaptic terminals, we depolymerized MTs by incubating slices with vinblastine at PT. The extent of MT depolymerization by vinblastine was assayed in living samples by real-time monitoring SiR-tubulin fluorescence intensity (Lukinavičius et al., 2014; Guillaud et al., 2017). After applying SiR-tubulin to a bath solution, tubulin polymers labeled with SiR were clearly visible throughout the terminals (Fig. 3*A*). During the incubation of slices with vinblastine (50 μm), SiR-tubulin fluorescence in calyceal terminals gradually declined in intensity. After 20 min of treatment with vinblastine (50 μm), SiR-tubulin fluorescence intensity decreased by ∼30%, whereas it remained unchanged in untreated controls (Fig. 3*B*). Longer treatment further reduced the fluorescence intensity by up to 90% after 120 min of incubation. The depolymerizing effect of vinblastine was dose dependent (1–50 μm, Fig. 3*C*). The MT-specific depolymerizing effect of vinblastine treatment was confirmed in a separate set of experiments using HeLa cells, where vinblastine (50 μm) treatment reduced SiR-tubulin fluorescence, but had no effect on SiR-actin fluorescence, whereas the latter was reduced by incubation with latrunculin A (20 μm; data not shown). Further, to confirm the extent of MT-depolymerization by vinblastine, we measured the fluorescence intensities of tubulin isoforms in calyceal terminals in slices fixed with paraformaldehyde after 60 min vinblastine treatment (50 μm) and immunostained with α- or β3-tubulin antibody (Fig. 3*D*). After vinblastine treatment, both α- and β3-tubulin fluorescence intensity declined by 30–40%, whereas VGluT1 fluorescence remained unchanged (Fig. 3*E*).

### Depolymerization of microtubules or F-actin had no effect on basal synaptic transmission or exoendocytosis of synaptic vesicles

**Figure 4.**
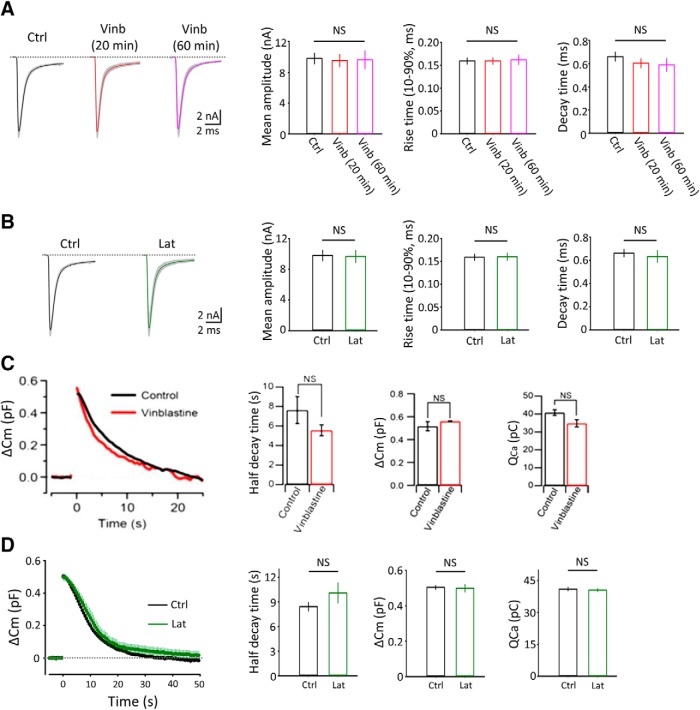
No significant effect of vinblastine or latrunculin A treatment on basal synaptic transmission and exocytosis-endocytosis of synaptic vesicles. ***A***, EPSCs with or without vinblastine treatment (50 μm, 20 or 60 min). Sample traces, control EPSCs without vinblastine treatment, and those after 20 (red) or 60 (pink) min vinblastine treatment. Bar graphs indicate mean amplitude (left), 10–90% rise time (middle), and decay time constant (right) of EPSCs without vinblastine (black) or 20 or 60 min after vinblastine treatment. There was no significant difference in these parameters of EPSCs between vinblastine-treated and untreated slices (one-way ANOVA: *F*_(2,24)_ = 0.76, *p* = 0.09). ***B***, EPSCs with or without latrunculin A treatment (20 μm, 60 min). Bar graphs indicate mean amplitude (left), 10–90% rise time (middle), and decay time constant (right) of EPSCs with (green) or without (black) latrunculin A treatment. No significant difference. ***C***, Membrane capacitance changes (Δ*C*_m_) induced by Ca^2+^ currents (data not shown) evoked in calyceal terminals at PT in slices treated with vinblastine (50 μm, 30–60 min at PT, *n* = 5 terminals) with (red trace) or without (*n* = 4, black trace) vinblastine treatment (superimposed). Bar graphs summarize endocytic half-decay time (left), exocytic magnitude (Δ*C*_m_, middle), and *Q*_ca_ (right) with or without vinblastine treatment. No significant difference with *p* = 0.25, 0.37, and 0.054 for endocytic half-decay time, exocytic magnitude, or Ca^2+^ current charge, respectively. ***D***, Membrane capacitance changes in slices treated with latrunculin A (20 μm, 60 min at PT, *n* = 6 terminals) with (green) or without (*n* = 7 terminals, black) latrunculin A treatment (superimposed). Bar graphs summarize endocytic half-decay time (left), exocytic magnitude (Δ*C*_m_, middle), and *Q*_ca_ (right) with or without latrunculin A treatment. No significant difference with *p* = 0. 07, 0.35, and 0.12, respectively, for endocytic decay time, exocytic magnitude, and Ca^2+^ current charge (two-tailed unpaired *t* test). NS, No significant difference; Ctrl, Control; Vinb, vinblastine; Lat, latrunculin A. Error bars indicate ± SEM.

Using vinblastine, we first examined whether MTs might be involved in the regulation of basal synaptic transmission at PT (Fig. 4*A*). After vinblastine treatment (50 μm) for 20–60 min, neither mean amplitude nor rise-time kinetics of EPSCs was changed. The decay time constant of EPSCs also remained unchanged. Likewise, latrunculin A treatment for 60 min had no effect on the amplitude or kinetics of EPSCs (Fig. 4*B*). Neither the frequency nor the amplitude of miniature EPSCs was affected by vinblastine or latrunculin treatment (data not shown).

We next examined whether depolymerization of MTs or F-actin might affect SV exocytosis or endocytosis, using membrane capacitance measurements at the calyx of Held presynaptic terminals (Sun et al., 2002; Yamashita et al., 2005). Exocytic-endocytic changes of membrane capacitance were induced by presynaptic Ca^2+^ currents elicited by a 20 ms pulse stepped from −80 to 10 mV at PT. Vinblastine treatment (50 μm, 30–60 min) had no effect on Ca^2+^ current charge (*Q*_Ca_), magnitude of exocytosis (Δ*C*_m_), or endocytic kinetics (Fig. 4*C*). Likewise, latrunculin A treatment (20 μm, 60 min) had no effect on *Q*_Ca_ or SV exocytosis-endocytosis (Fig. 4*D*). Thus, depolymerization of MTs or F-actin had no effect on the exocytosis-endocytosis of synaptic vesicles or basal synaptic transmission at the calyx of Held.

**Figure 5.**
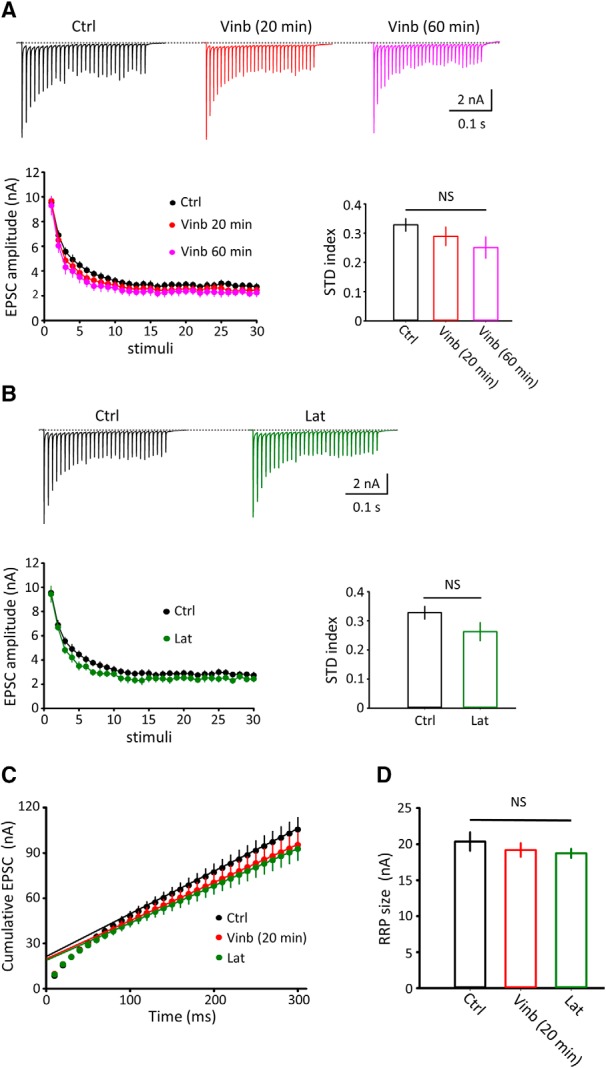
Depolymerization of MTs or F-actin had no effect on STD, but differentially prolonged the recovery of EPSCs from STD. ***A***, Top traces, STD induced by a train of 100 Hz (×30) stimulation, with (red) or without (black) vinblastine treatment (50 μm, 20 or 60 min). Time plots in bottom left show STD with or without vinblastine treatment (superimposed). Bar graphs indicate the magnitude of STD measured by averaging the last 10 EPSC amplitudes divided by the initial EPSC amplitude in the train. No significant difference between them (NS, one-way ANOVA: *F*_(2,24)_ = 1.35, *p* = 0.12). ***B***, As in ***A***, except that slices were treated with latrunculin A (20 μm, 60 min) instead of vinblastine. No significant difference (*p* = 0.08, two-tailed unpaired *t* test). ***C***, Cumulative EPSC amplitude plots during a 100 Hz train in control (Ctrl; black), vinblastine (Vinb; 50 μm, 20 min, red), latrunculin A (Lat; 20 μm, 60 min; green). The last 10 data points were used for fitting with linear regression lines (dashed lines) and extrapolated to 0 ms to estimate the RRP size of synaptic vesicles. ***D***, Mean RRP size estimated in Ctrl (20.4 ± 0.98 nA, *n* = 10), Vinb (19.2 ± 0.96 nA, *n* = 10), Lat (18.7 ± 0.82 nA, *n* = 7). There was no significant difference (NS) among them (one-way ANOVA: *F*_(3,28)_ = 0.88, *p* = 0.46). Error bars indicate ± SEM.

Like basal synaptic transmission, STD induced by a brief (0.3 s) train of stimulation at high frequency (100 Hz) was not significantly affected by vinblastine (Fig. 5*A*; *p* = 0.1, *n* = 10) or latrunculin A (Fig. 5*B*; *p* = 0.08, *n* = 7), although the STD magnitude showed a tendency to increase after their treatments. Likewise, the size of readily releasable vesicles (RRPs) estimated from cumulative EPSC amplitudes (Schneggenburger et al., 1999) showed no significant difference between slices in controls, after vinblastine treatment (20 min) or latrunculin A treatment (one-way ANOVA: *F*_(3,28)_ = 0.88, *p* = 0.46, *n* = 7–10; Fig. 5*C*,*D*).

### Depolymerization of microtubules or F-actin prolonged kinetics of recovery from STD

**Figure 6.**
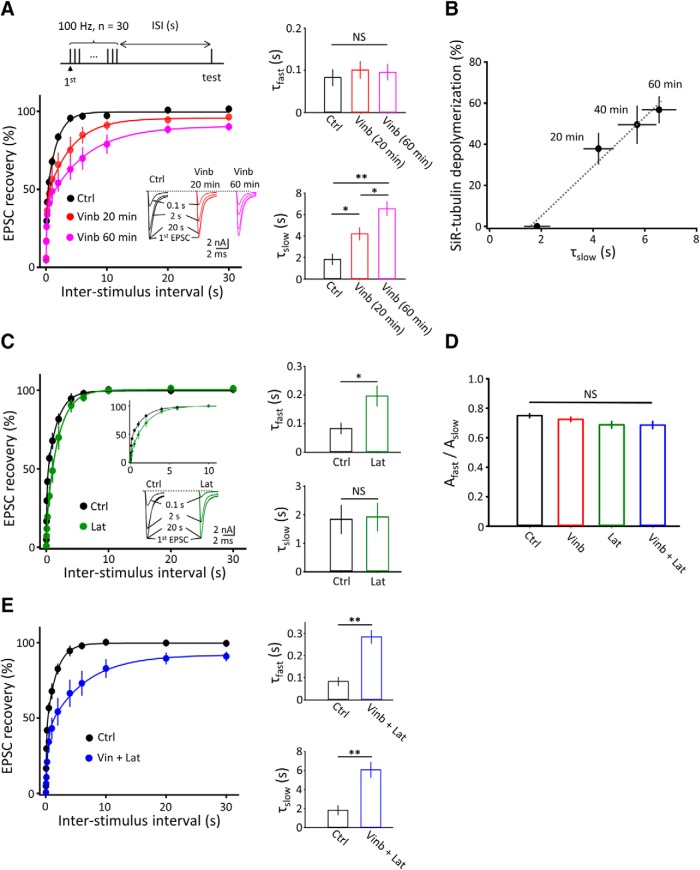
Depolymerization of MTs or F-actin differentially prolonged the recovery of EPSCs from STD. ***A***, Percentage recovery of EPSC amplitude from STD at different interstimulus intervals (stimulation protocol on the top). Sample traces of EPSCs at different interstimulus intervals (ISI) are shown in inset (superimposed); 20 min (red) or 60 min (pink) after vinblastine treatment (Vinb) or without vinblastine treatment (Ctrl; black). Bar graphs in top and bottom indicate τ_fast_ and slow τ_slow_, respectively, after 20 min (*n* = 10) or 60 min (*n* = 7) of treatment with vinblastine or without vinblastine treatment (*n* = 10). Vinblastine treatment specifically prolonged τ_slow_. *p < 0.05, **p < 0.01 (one-way ANOVA). ***B***, Correlation between the percentage of MT depolymerization (ordinate; data from Fig. 3*B*) and τ_slow_ values after different lengths of vinblastine treatment for 20 min (*n* = 10), 40 min (*n* = 5), and 60 min (*n* = 7). The slope coefficient (*r*) of a linear regression line is 0.98. ***C***, Latrunculin A treatment (Lat; 20 μm, 60 min; green) specifically prolonged τ_fast_ from STD, as shown with the expanded time scale in the top inset. EPSCs during recovery from STD without (black) or with latrunculin A (green) treatment are shown in the bottom inset (superimposed). Bar graphs indicate the τ_fast_ and τ_slow_ in top and bottom, respectively, with (green, *n* = 7) or without (black, *n* = 10) latrunculin A treatment. *p < 0.05 (two tailed unpaired *t* test). ***D***, Relative ratio of fast and slow components (A_fast_/A_slow_) estimated for Ctrl (0.75 ± 0.01, *n* = 10, black), Vinb (0.72 ± 0.02, *n* = 10, red), Lat (0.69 ± 0.02, *n* = 7, green), and Vinb (50 μm) + Lat (20 μm, 60 min; 0.69 ± 0.03, *n* = 5, blue). No significant difference (NS) among them (one-way ANOVA: *F*_(3,28)_ = 2.81, *p* = 0.06). ***E***, Cotreatment of slices with vinblastine (50 μm) and latrunculin A (20 μm) for 60 min (blue) prolonged the recovery of EPSCs from STD at both fast and slow recovery components (bar graphs). The magnitudes of prolongation of τ_fast_ and τ_slow_ are similar to those of individual drug treatments (two-tailed unpaired *t* test, *p* = 0.27). Error bars indicate ± SEM.

After STD, induced by a train of nerve stimulation (100 Hz, 0.3s) at PT, EPSCs underwent a biexponential recovery with a subsecond time constant (τ_fast_) followed by a second-order time constant (τ_slow_; Fig. 6*A*). Although vinblastine treatment for 20–60 min had no significant effect on the STD magnitude (Fig. 5*A*,*B*), it markedly and specifically prolonged the τ_slow_ (one-way ANOVA: *F*_(2,24)_ = 16.15, *p* < 0.01; *n* = 10 and 7, respectively, for 20 and 60 min of treatment) without affecting the τ_fast_ (one-way ANOVA: *F*_(2,24)_ = 0.24, *p* = 0.39). The magnitude of τ_slow_ prolongation by vinblastine showed a strong correlation (Pearson correlation coefficient, *r* = 0.98) with that of MT depolymerization, assayed using SiR-tubulin (Fig. 3*B*) during vinblastine treatment (Fig. 6*B*). In clear contrast to vinblastine, latrunculin A treatment (20 μm, 60 min) specifically prolonged the fast recovery time constant (two tailed unpaired *t* test: *p* < 0.05, *n* = 7 cells) with no effect on the slow time constant (Fig. 6*C*). The percentage of τ_fast_ relative to τ_slow_ was essentially the same with or without treatment with vinblastine or latrunculin A (Fig. 6*D*).

In various cell systems, MTs and F-actin reportedly cross talk functionally (Malorni et al., 2003; Etienne-Manneville, 2004; Arnette et al., 2016). To examine whether this might be the case for the recovery of EPSCs from STD, we cotreated slices with vinblastine and latrunculin A. After 60 min incubation of slices with vinblastine (50 μm) and latrunculin A (20 μm), recovery from STD was significantly prolonged, both at the fast and slow components (Fig. 6*E*), as expected from the summed effect of vinblastine or latrunculin A (Fig. 6*A*,*C*). Thus, MTs and F-actin differentially contribute to slow and fast recoveries of EPSCs from STD without cross talk.

### Physiological roles of MTs and F-actin in high-frequency neurotransmission

**Figure 7.**
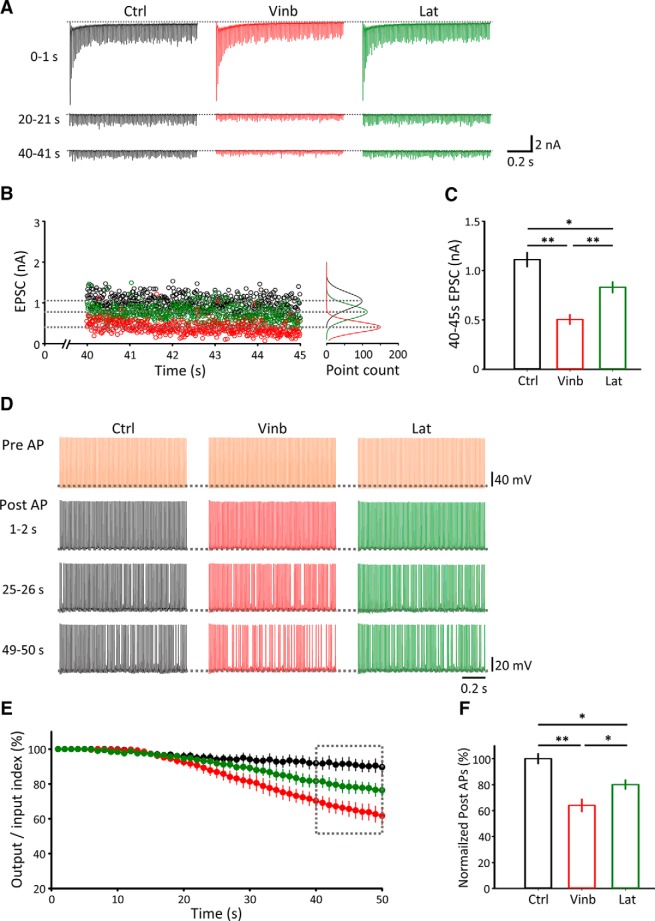
Presynaptic cytoskeletal filaments contribute to the maintenance of the fidelity of high-frequency neurotransmission. ***A***, EPSCs evoked at PT by a train of stimulation at 100 Hz at 0–1 s (top traces), 20–21 s (middle), and 40–41 s (bottom) in control (Ctrl; black), after vinblastine treatment (Vinb; 50 μm; red), or latrunculin A treatment (Lat; 20 μm, 60 min; green). Stimulation artifacts are truncated. ***B***, EPSC amplitudes at 40–45 s with their distributions (right graphs). ***C***, Bar graphs of mean EPSC amplitudes at 40–45 s in Ctrl (1.11 ± 0.07 nA, *n* = 12, black), Vinb (0.5 ± 0.02 nA, *n* = 10, red), and Lat (0.83 ± 0.05 nA, *n* = 8, green). Significant difference in EPSC amplitudes among them (*F*_(2,27)_ = 20.04, *p* < 0.01, one-way ANOVA). *p < 0.05, **p < 0.01. ***D***, Presynaptic APs elicited at 100 Hz at PT (top raw traces). Postsynaptic APs evoked by presynaptic APs at 1–2 s (second raw trace), 25–26 s (third raw trace), and 49–50 s (bottom raw trace) after stimulation onset, without drug treatment (left columns, control; black traces), after vinblastine treatment (50 μm, 20 min, middle columns; red), or after latrunculin A treatment (20 μm, 60 min, right columns; green). ***E***, The fidelity of neurotransmission evaluated from the ratios of output AP number/input AP number (ordinate) in controls (black), after vinblastine treatment (red), or after latrunculin A treatment (green). ***F***, Bar graphs indicating the fidelity of neurotransmission at 40–50 s after 100 Hz stimulation onset. Significant difference (one-way ANOVA: *F*_(2,14)_ = 18.51, *p* < 0.01) in transmission fidelity between vinblastine-treated samples (*n* = 6) and controls (*n* = 6; one-way ANOVA with *post hoc* Scheffé's tests, *p* < 0.01), between latrunculin A-treated samples (*n* = 5) and controls (*n* = 6; one-way ANOVA with *post hoc* Scheffé's tests, *p* < 0.05), and between vinblastine-treated samples (*n* = 6) and latrunculin A-treated samples (*n* = 5; one-way ANOVA with *post hoc* Scheffé's tests, *p* < 0.05). *p < 0.05, **p < 0.01. Error bars indicate ± SEM.

Depolymerization of MTs or F-actin had no significant effect on basal synaptic transmission (Fig. 4) or STD (Fig. 5), but significantly prolonged the recovery of EPSCs from STD (Fig. 6). These results suggest that cytoskeletal filaments may play essential roles in the maintenance of long-lasting high-frequency neurotransmission. To test this, we first evoked EPSCs at 100 Hz for 45 s (Fig. 7*A*). EPSCs continuously underwent a depression during stimulation, and the magnitude of depression at 40–45 s was significantly greater in slices treated with vinblastine (one-way ANOVA: 50 μm, 20 min; *n* = 10, *p* < 0.01) or latrunculin A (one-way ANOVA: 20 μm, 60 min; *n* = 8, *p* < 0.05) relative to controls (*n* = 12; Fig. 7*B*,*C*).

To evaluate the impact of EPSC depression on synaptic neurotransmission, we next made simultaneous whole-cell recordings of presynaptic and postsynaptic APs, elicited by a presynaptic stimulation at 100 Hz at PT (Fig. 7*D*). Compared with similar experiments at RT (Eguchi et al., 2017), the fidelity of neurotransmission (post-AP/pre-AP) remained higher, close to 100% for 50 s, at PT (Fig. 7*E*,*F*). Vinblastine treatment (50 μm, 20 min) impaired the fidelity of neurotransmission by increasing the number of AP failures in postsynaptic neurons (*n* = 6, *p* < 0.01, one-way ANOVA). Latrunculin A treatment (20 μm, 60 min) also impaired the fidelity (*n* = 5, *p* < 0.05, one-way ANOVA), but to a lesser extent than vinblastine treatment (Fig. 7*E*,*F*). These results suggest that presynaptic cytoskeletal filaments, MTs and F-actin, significantly contribute to the maintenance of high-frequency neurotransmission.

## Discussion

The presence and functional role of MTs in the presynaptic terminal have been a matter of debate (Bodaleo and Gonzalez-Billault, 2016). In the calyx of Held terminals of rat brainstem, we confirmed our previous observation in cultured calyceal terminals (Guillaud et al., 2017) that axonal MTs are extended into terminal swellings and further found, using STED microscopy, that they are partially colocalized with SVs. These results agree with previous EM studies indicating the presence of tubulin polymers at the frog NMJ (Gray, 1978, 1983; Hirokawa et al., 1989).

To address whether MTs play a role in synaptic transmission, we depolymerized MTs using vinblastine after estimating its potency using SiR-tubulin live imaging. When MTs in calyceal terminals were depolymerized with vinblastine, the recovery of EPSCs from STD was prolonged, exclusively at the slow component of biexponential recovery. This slow component was resistant to F-actin depolymerization by latrunculin A (Fig. 6*C*) as previously reported (Sakaba and Neher, 2003), and it is distinct from the recovery of the slow releasing pool (Sakaba and Neher, 2001). The time constant of this slow recovery component (∼2 s at PT) is much faster than that of SV refilling with transmitter glutamate (∼7 s at PT; Hori and Takahashi, 2012), suggesting that it reflects the replenishment of SVs already filled with transmitter from reserve pool (RP) to RRP. Vinblastine slowed SV replenishment from RP to RRP (Fig. 6*A*) but did not significantly affect RRP size during a brief train stimulation (Fig. 5*C*,*D*). However, during a long train of stimulation, vinblastine significantly depressed EPSC amplitude presumably because of RRP depletion (Fig. 7*A–C*), thereby blocking neurotransmission (Fig. 7*D–F*).

The molecular mechanism underlying this slow recovery component is unknown, except for its dependence on GTP (Takahashi et al., 2000). Several possibilities may be conceived for the role of MTs in this SV transport. First, MTs may directly transport SVs to release sites, although MTs are selectively involved in long-distance SV movements in cultured calyceal terminals (Guillaud et al., 2017). Second, mitochondria, which are transported along MTs (Hirokawa et al., 2010; Melkov and Abdu, 2018), may provide ATP close to SVs to promote their movement. However, this is unlikely since, during simultaneous presynaptic and postsynaptic AP recording (Fig. 7*D–F*), the presynaptic pipette contained 3 mm ATP, which will hold intraterminal ATP concentration (Pusch and Neher, 1988), but vinblastine impaired transmission fidelity. In fact, in cultured hippocampal synapses, ATP concentration in presynaptic boutons is reportedly uniform between boutons with or without mitochondria, implying that ATP rapidly diffuses through axons (Pathak et al., 2015). Third, SVs tethered to MTs in the terminal (Hirokawa et al., 1989) may serve as a reserve pool for SV reuse, and tethered SVs may be scattered after MT depolymerization, thereby reducing the number of SVs available for recycling reuse. The molecular mechanism of this SV transport underlying the recovery of transmission after STD remains to be studied.

As reported previously at the calyx of Held at RT (Sakaba and Neher, 2003; Lee et al., 2012) and at cerebellar synapses at or near PT (Miki et al., 2016), the fast component of recovery from STD was prolonged by F-actin depolymerization with latrunculin at the calyx of Held at PT (Fig. 6*C*). At this synapse at RT, the fast recovery component is clearly observed when a large Ca^2+^ influx was induced, by high-frequency stimulation (Wang and Kaczmarek, 1998, 100 Hz, 0.5 s; Lipstein et al., 2013, 100–300 Hz, 0.1 s) or by presynaptic depolarization under voltage clamp with a square pulse (Sakaba and Neher, 2001; Lipstein et al., 2013; 50 ms) in the presence of K^+^ channel blockers, whereas it is absent when EPSCs were evoked at low frequency (10 Hz; Takahashi et al., 2000) or at high frequency, but without K^+^ channel blockers (Wang and Kaczmarek, 1998, 100 Hz; Mahfooz et al., 2016, 300 Hz). At PT, however, the fast recovery component was prominent after 100 Hz stimulation in the absence of K^+^ channel blocker (Fig. 6). The fast recovery component after STD reportedly depends on Ca^2+^ (Wang and Kaczmarek, 1998), calmodulin (Sakaba and Neher, 2001), Munc13–1 (Lipstein et al., 2013), as well as F-actin (Sakaba and Neher, 2003) and intersectin (Sakaba et al., 2013), and it is abolished when endocytosis is blocked (Hosoi et al., 2009). The fast recovery component is thought to reflect superpriming of SVs (Lee et al., 2012; Miki et al., 2016) through release site clearance (Hosoi et al., 2009; Hua et al., 2013) or/and fast SV replenishment (Lipstein et al., 2013).

Various roles of F-actin in the nerve terminal have been reported. A negative regulatory role of F-action on exocytic SV fusion is reported at cultured hippocampal synapses (Morales et al., 2000), but, at the calyx of Held, F-actin depolymerization had no effect on the magnitude of SV exocytosis or EPSC amplitude (Fig. 4). Genetic ablation of F-actin reportedly blocks both clathrin-dependent and -independent endocytosis (Wu et al., 2016). Pharmacological experiments can lead to controversial results due to the variable accessibility of drugs to targets, whereas the gene knock-out approach has disadvantages of compensatory and secondary effects (Wu et al., 2016). In the present study, pharmacological depolymerization of F-actin by latrunculin A prolonged the fast-recovery component after STD (Fig. 6*C*) and impaired fidelity of high-frequency transmission (Fig. 7), indicating that the drug was accessible to the presynaptic target. Nevertheless, latrunculin A treatment had no effect on SV endocytosis at the calyx of Held at PT (Fig. 4*D*) as well as at RT (Eguchi et al., 2017). More extensive F-actin disassembly might be necessary for blocking SV endocytosis.

Pharmacological depolymerization of F-actin is reported to block ultrafast endocytosis of the subsecond time course (Watanabe et al., 2013), which operates preferentially at PT (Watanabe et al., 2014; Delvendahl et al., 2016). Ultrafast endocytosis takes tens of seconds to reform SVs from endosomes to be reused (Soykan et al., 2017; Watanabe et al., 2018), but it may also contribute to release site clearance, like clathrin-mediated endocytosis (Hosoi et al., 2009; Lee et al., 2012). However, capacitance measurements at the calyx of Held at PT indicated that latrunculin A treatment had no appreciable effect on SV exocytosis-endocytosis (Fig. 4*D*), suggesting that ultrafast endocytosis unlikely operates significantly at the calyx of Held.

High-precision, high-frequency neurotransmission plays pivotal roles in various brain functions, such as sensory processing, cognition, memory formation (Sabatini and Regehr, 1999; Buzsáki and Draguhn, 2004; Uhlhaas and Singer, 2010), and motor control functions (Sugihara et al., 1993). Marked prolongation of recovery from STD and impairments in the fidelity of high-frequency neurotransmission after MT depolymerization (Fig. 6) suggest that presynaptic MTs normally accelerate SV recycling, thereby contributing to the maintenance of integrative synaptic functions.
